# Extracellular Lipopolysaccharide Triggers the Release of Unconjugated Interferon-Stimulated Gene 15 (ISG15) Protein from Macrophages via Type-I Interferon/Caspase-4/Gasdermin-D Pathway

**DOI:** 10.3390/pathogens15010122

**Published:** 2026-01-22

**Authors:** Sudiksha Pandit, Lindsay Grace Miller, Indira Mohanty, Santanu Bose

**Affiliations:** Department of Veterinary Microbiology and Pathology, College of Veterinary Medicine, Washington State University, Pullman, WA 99164, USA

**Keywords:** Interferon-stimulated gene 15, lipopolysaccharide, type-I interferon, human macrophages, caspase-4, Gasdermin D

## Abstract

Interferon-stimulated gene 15 (ISG15) is an interferon-induced ubiquitin-like protein that plays an important role in antiviral defense and inflammatory responses, primarily through the process of ISGylation, whereby ISG15 is covalently conjugated to target proteins. Beyond its intracellular functions, a portion of free unconjugated ISG15 is also released into the extracellular environment during infections and diseases such as cancer. Extracellular ISG15 is known to regulate immune cell activity and cytokine production. Despite its immune-modulatory role, how ISG15 is released from cells has remained unclear. In this study, we have identified a non-lytic mechanism by which human macrophages release ISG15. Using lipopolysaccharide (LPS) as a stimulus, we show that extracellular LPS triggers unconjugated ISG15 release by utilizing plasma membrane-localized Gasdermin D (GSDMD) pores. Mechanistically, LPS via the autocrine/paracrine action of type-I interferon (IFN) activates caspase-4 (Casp4) to cleave the N-terminal domain of GSDMD for the formation of cell surface GSDMD pores that permit the extracellular release of unconjugated ISG15 in the absence of lytic cell death. Together, our studies have identified the IFN-Casp4-GSDMD axis as a previously unrecognized non-classical pathway for unconjugated ISG15 release from cells.

## 1. Introduction

Interferon-stimulated gene 15 (ISG15) plays a key role in various biological functions, including host defense against pathogens and modulating cellular response during various diseases like cancer, cardiovascular disorders, and neurological diseases [1,2,3,4,5,6,7,8]. ISG15 mediates its effect by facilitating the post-translational modification known as ISGylation [9,10]. During ISGylation, ISG15 is covalently conjugated to a wide spectrum of proteins by cellular conjugating enzymes [10,11,12,13,14]. ISGylated proteins are involved in regulating various cellular/biological functions [4,15]. Although ISGylation is an intracellular (cytoplasmic) event, cells are capable of releasing ISG15 in its unconjugated, free form in response to various physiological and pathological conditions [2,16,17,18,19,20,21,22,23]. For example, an infection with viruses (influenza A virus, rhinovirus, SARS-CoV-2, Zika virus) triggers ISG15 release from infected cells [24,25,26]. Of particular significance, the presence of extracellular ISG15 has been observed in clinical samples such as the serum of patients infected with rhinovirus and SARS-CoV-2 [25,26]. Interestingly, rhinovirus load and symptom score were positively correlated with the extracellular ISG15 levels detected in the nasal and saliva secretions of virus-infected patients [25]. Therefore, extracellular ISG15 detected during an infection may modulate immune response to dictate infection severity and disease outcome. During rhinovirus infection, extracellular ISG15 released from infected epithelial cells modulated the production of CXCL10, a key chemokine regulating immunity [25]. Likewise, ISG15 released by SARS-CoV-2-infected macrophages triggers a pro-inflammatory response by inducing cytokines like IL-6 and IL-1β, which contribute to the cytokine storm that determines the severity of the virus-associated disease [26,27]. Furthermore, a link between ISG15 deficiency and susceptibility to Mycobacterial Disease (MSMD) has been reported [28]. MSMD patients failed to release ISG15, and as a result these patients exhibited an impaired induction of interferon-γ, an essential cytokine controlling mycobacterial infections [28]. Additionally, extracellular ISG15 plays an important immune-modulatory role in immune cells, since it stimulates the production of important immune cytokine IFN-γ from PBMCs, NK cells, and T cells [28,29,30,31,32,33,34].

Although released (extracellular) unconjugated ISG15 plays a role in regulating the immune response, the mechanism by which ISG15 is released from cells is still unknown. Particularly, it is important to mention that ISG15 does not possess a signal peptide, and therefore it is not released by the classical protein secretory pathway [33]. Furthermore, since ISG15 release has been noted at time-points when cells are not undergoing lytic cell death [26], a non-lytic cellular mechanism may exist to facilitate the extracellular release of unconjugated ISG15. In the current study, by using lipopolysaccharide (LPS) as an ISG15 release triggering agent, we have identified the autocrine/paracrine action of the type-I interferon (IFN) in promoting ISG15 release from LPS-primed macrophages through the activated caspase-4 (Casp4)-mediated cleavage of Gasdermin D (GSDMD), which forms plasma membrane pores to release ISG15 to the extracellular milieu. Thus, the IFN/Casp4/GSDMD pathway represents a non-lytic cellular mechanism involved in ISG15 release from macrophages.

LPS is an endotoxin which constitutes the major cell wall component of clinically important Gram-negative bacteria such as *Escherichia coli*, *Klebsiella pneumoniae*, and *Pseudomonas aeruginosa* [35,36] LPS acts as a PAMP (Pathogen-Associated Molecular Pattern) to activate PRR (Pattern Recognition Receptor) TLR4 for triggering a “massive” inflammatory immune response that results in septic shock (sepsis) and death [37,38]. Thus, LPS-mediated unchecked exaggerated immune response contributes to sepsis. Previous studies have shown LPS promoting ISGylation in mouse macrophages [39]. However, whether LPS triggered ISG15 release was unknown. Our current study demonstrated that the extracellular LPS treatment of human macrophages induces ISGylation and ISG15 release. Furthermore, we show that the autocrine/paracrine action of IFN-promoting ISG15 release from extracellular LPS-primed non-lytic macrophages by utilizing the Casp4/GSDMD pathway. Thus, our identification of the IFN/Casp4/GSDMD cellular pathway constitutes the first report of a non-lytic cellular mechanism involved in the extracellular release of unconjugated free ISG15 from cells.

## 2. Materials and Methods

### 2.1. Cell Culture

Human THP-1 monocytic cell-line (ATCC, Manassas, VA, USA; catalog number—TIB-202) was cultured in RPMI-1640 medium (Gibco-ThermoFisher Scientific, Waltham, MA USA, catalog number—11875093) supplemented with 10% fetal bovine serum (FBS), 100 IU/mL penicillin, 100 µg/mL streptomycin, 1 mM sodium pyruvate, 10 mM HEPES [4-(2-hydroxyethyl)-1-piperazineethanesulfonic acid], and 50 µM β-mercaptoethanol (Sigma-Aldrich, St. Louis, MO, USA). THP-1 cells were seeded in 12-well or 48-well plates at a density of 1 × 10^6^ cells/well and 2 × 10^5^ cells/well, respectively. Cells were differentiated by treating them with 100 nM phorbol 12-myristate 13-acetate (PMA) (Sigma-Aldrich) for 24 h. Undifferentiated and non-adherent cells were removed by washing with phosphate-buffered saline (PBS), and the adherent cells were incubated for an additional 24 h in PMA-free medium before further treatments.

### 2.2. Cell Treatment

Differentiated THP-1 cells were treated with either lipopolysaccharide (LPS, 1 µg/mL) (InvivoGen, San Diego, CA, USA, catalog number—tlrl-3pelps) or interferon-β (IFN-β, 1000 U/mL) (Sino Biological, Beijing, China, catalog number—10704-HNAS) for the indicated time points.

Cells were pre-treated for 2 h with reconstituted vehicle or specific inhibitors, including Disulfiram (30 µM; MedChemExpress, Monmouth Junction, NJ, USA, catalog number—HY-B0240), Ac-LEVD-CHO (4 µM; Santa Cruz Biotechnology, catalog number—396109), recombinant B18R protein (0.25 µg/mL; InvivoGen, catalog number—inh-b18r), TAK-242 (1 µM; MedChemExpress, catalog number—HY-11109), Ruxolitinib (1 µM; MedChemExpress, catalog number—HY-50856), and 3-Methyladenine (3MA) (10 µM; Sigma-Aldrich, catalog number—5142-23-4). Following pre-treatment, cells were treated with either LPS (0.5 µg/mL) or IFN-β (500 IU/mL) for the indicated times. In several experiments, Dimethyl sulfoxide (DMSO) (Sigma Aldrich, catalog number—D2650) was used as a vehicle control.

### 2.3. Western Blotting

Whole-cell lysates (WCLs) were collected using 1× sample buffer containing β-mercaptoethanol, followed by sonication to ensure complete disruption. The lysates were heated at 100 °C for 5 min and then spun briefly at 13,000 rpm to clear insoluble material. The resulting samples were separated by SDS-PAGE on an acrylamide gel and transferred onto 0.2 µm nitrocellulose membranes (Amersham Protran-Cytiva, Marlborough, MA, USA). Membranes were blocked with 5% milk in PBS-T (0.1% Tween-20 in PBS) for 5 h at room temperature and incubated overnight at 4 °C with primary antibodies, including ISG15 antibody (affinity-purified polyclonal antibody as described previously [9], Gasdermin D antibody (Cell Signaling Technology, Danvers, MA, USA, catalog number—39754S) and actin antibody (Bethyl Laboratories, Montgomery, TX, USA, catalog number—A300-491A). The following day, membranes were washed and treated with horseradish peroxidase-conjugated secondary antibodies for 1.5 h at room temperature. The Western Lightning Plus-ECL chemiluminescence system (PerkinElmer, Waltham, MA, USA, catalog number—104001EA) was used to detect protein bands.

### 2.4. TCA Precipitation

To assess extracellular ISG15, medium supernatants were collected and cleared by centrifugation at 2000 rpm for 10 min to remove cellular debris. Proteins in the supernatants were precipitated by adding trichloroacetic acid (TCA) to a final concentration of 20% (*v/v*) and incubating overnight at 4 °C. The resulting protein pellets were washed three times with ice-cold acetone, air-dried briefly, and then resuspended in 2× sample buffer supplemented with β-mercaptoethanol. Samples were neutralized using 1 M Tris-HCl (pH 8.0) prior to Western blotting analysis with the appropriate antibodies.

### 2.5. Lactate Dehydrogenase (LDH) Assay

Medium supernatants were collected from treated cells. The supernatants were centrifuged at 8000 rpm for 10 min to remove debris prior to analysis. LDH levels in the cleared supernatants were measured using the LDH Reaction Mix (abcam, Waltham, MA, USA, catalog number—ab65393) according to the manufacturer’s instructions. Absorbance was measured at 450 nm using a microplate reader, and percent cytotoxicity was calculated according to the manufacturer’s instructions. Cells treated with a supplier-provided cytotoxicity-inducing reagent served as a positive control.

### 2.6. Immunoprecipitation

Cells were treated with vehicle or LPS (1 µg/mL) for 24 h. Medium supernatants were collected and centrifuged at 5000 rpm for 10 min at 4 °C to remove debris. Protein G agarose beads (ThermoFisher Scientific, Waltham, MA USA, catalog number—20398) were incubated with ISG15 antibody overnight at 4 °C to allow antibody conjugation. The clarified supernatants were then incubated with ISG15 antibody-conjugated beads overnight at 4 °C. Following incubation, the bead–supernatant mixture was centrifuged, and the supernatant was discarded. The beads were washed ten times with wash buffer (1× PBS, 10 mM Tris-HCl [pH 7.4], and 1× protease inhibitor). Bound proteins were eluted by boiling the beads in 2× sample buffer. Eluted samples were analyzed through Western blotting using an anti-ISG15 antibody.

## 3. Results

### 3.1. Extracellular LPS Triggers ISGylation and ISG15 Release in Human THP-1 Macrophages

ISGylation in LPS-treated mouse macrophages has been reported [39]. However, it is unknown whether it also occurs in human macrophages. Furthermore, whether LPS-stimulated cells release ISG15 is also unknown. Therefore, we first investigated ISGylation status in LPS-stimulated human macrophages.

**Figure 1:**
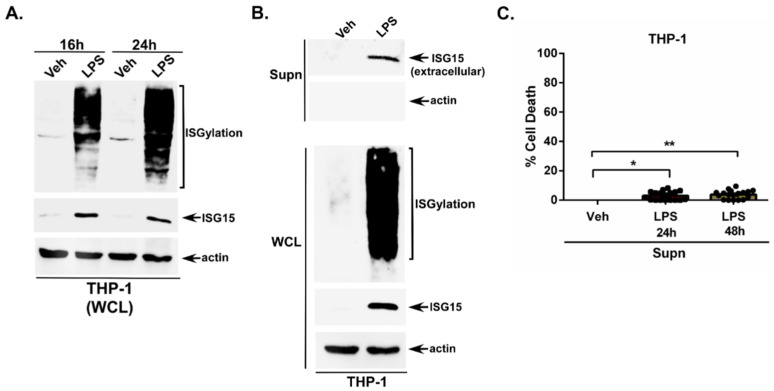
Extracellular LPS triggers ISGylation and ISG15 release from human macrophages. (**A**) Human THP-1 macrophages were treated with either vehicle control (Veh) or LPS (1 µg/mL) for the indicated times, and whole-cell lysate (WCL) was subjected to Western blotting with ISG15 and actin antibodies to detect ISGylation and intracellular unconjugated ISG15 protein. (**B**) Human THP-1 macrophages were treated with either vehicle control (Veh) or LPS (1 µg/mL). WCL was subjected to Western blotting with ISG15 and actin antibodies to detect ISGylation and intracellular unconjugated ISG15 and actin proteins. The medium supernatants (supn) from the same experiment were precipitated with TCA, and total protein was subjected to Western blotting with ISG15 and actin antibodies to detect the presence of ISG15 and actin proteins in the extracellular milieu. (**C**) Medium supernatants from LPS-treated THP-1 macrophages (24 h and 48 h post-treatment) were used to quantify cell death through a lactate dehydrogenase (LDH) assay. The Western blots are representative of three independent experiments with similar results. The LDH assay values represent ± s.e.m; * *p* & ** *p* < 0.05 using a Student’s *t*-test.

Human THP-1 macrophages were treated with LPS extracellularly for 16 h and 24 h. The cell lysate was then subjected to Western blotting with an ISG15 antibody. We observed a robust ISGylation and ISG15 expression in LPS-treated THP-1 macrophages (Figure 1A). LPS-mediated ISGylation and ISG15 expression was also detected at 48 h post-LPS treatment (Figure S1A).

Since LPS induced intracellular ISG15 expression, we next examined whether unconjugated free ISG15 is released from LPS-treated cells. So far, such phenomena have not been examined in LPS-stimulated cells. For these studies, medium supernatant from LPS-treated THP-1 macrophages was collected to assess the presence of extracellular ISG15 through TCA precipitation and Western blotting with an ISG15 antibody. We utilized the TCA method to recover total extracellular ISG15 protein irrespective of its oligomerization (and complex with other proteins) status. Thus, the TCA method was a rational approach since unconjugated ISG15 has been reported to undergo homo-oligomerization and can be detected as homodimers [22,40].

Our results revealed that extracellular LPS can trigger ISG15 release from cells, since we detected unconjugated free ISG15 in the medium supernatant of THP-1 macrophages treated with LPS for 24 h (Figure 1B) and 48 h (Figure S1B). Please note that our TCA-precipitated medium supernatants are devoid of intracellular factors since Western blotting with an actin antibody revealed the absence of actin in these samples (Figure 1B), whereas Western blotting of the whole-cell lysate (WCL) collected from the same experiment demonstrated the presence of intracellular proteins such as actin (Figure 1B). We also detected ISGylation and ISG15 in the WCL subjected to Western blotting with the ISG15 antibody (Figure 1B). Thus, the extracellular ISG15 we detected in LPS-treated cells was released by a non-lytic mechanism, since actin was not detected in the medium supernatant and extracellular ISG15 was only detected in LPS-treated cells, corroborating with the detection of higher levels of intracellular (cell lysate) unconjugated ISG15 protein in LPS-treated, but not in vehicle-treated macrophages (Figure 1B). Importantly, the LDH cytotoxicity assay confirmed lytic cell death or the cellular cytotoxicity-independent release of ISG15 from LPS-treated macrophages, since we observed minimal (2–4%) cell death in macrophages treated with LPS for 24 h and 48 h (Figure 1C). Thus, our results specifically show that ISG15 detected in the medium supernatant constitutes extracellular ISG15 that is released from LPS-primed non-lytic cells.

Furthermore, ISG15 release from LPS-primed cells occurred via TLR4 activation since treating THP-1 cells with a non-cytotoxic dose of the widely used TLR4 inhibitor TAK-242 [41] abrogated ISG15 release from LPS-treated macrophages (Figure S1C). In order to investigate whether extracellularly released ISG15 is associated with exosomes/extracellular vesicles, we performed an immunoprecipitation analysis of the medium supernatant with an ISG15 antibody. The rationale for this experiment is that, if ISG15 is a component of exosomes/extracellular vesicles, it cannot be immunoprecipitated due to its location inside these extracellular structures. Our analysis revealed the presence of high levels of exosome/extracellular vesicle-free unconjugated free ISG15 in the extracellular milieu, since the ISG15 antibody readily precipitated ISG15 protein from the medium supernatant of LPS-primed cells (Figure S1D).

Thus, we show extracellular LPS possessing a pro-ISGylation activity in human macrophages. More importantly, we have also identified LPS as a new PAMP involved in the extracellular release of unconjugated free ISG15 protein.

### 3.2. LPS-Mediated ISG15 Release from Macrophages Is Gasdermin D Dependent

Gasdermin D (GSDMD) pores on the cell surface can shuttle various intracellular proteins into the extracellular environment both during cellular lytic and non-lytic conditions [42]. Both human/murine caspase-1 and human caspase-4 and -5 (caspase-11 is the murine homolog of caspase-4) cleave GSDMD via canonical and non-canonical inflammasome pathways, respectively [42,43]. The caspase-dependent cleavage of GSDMD generates N-terminal fragments that oligomerize to form pores in the plasma membrane. These plasma membrane pores are utilized to shuttle ions and host factors like cytokines from the cytosol to the extracellular milieu [42]. This process operates in intact non-lytic cells prior to lytic cell death (pyroptosis) from osmotic cell swelling and lysis due to ionic imbalance. Thus, GSDMD pores are involved in unconventional protein release either prior to or independent of cell lysis that occurs during pyroptosis [44,45].

**Figure 2:**
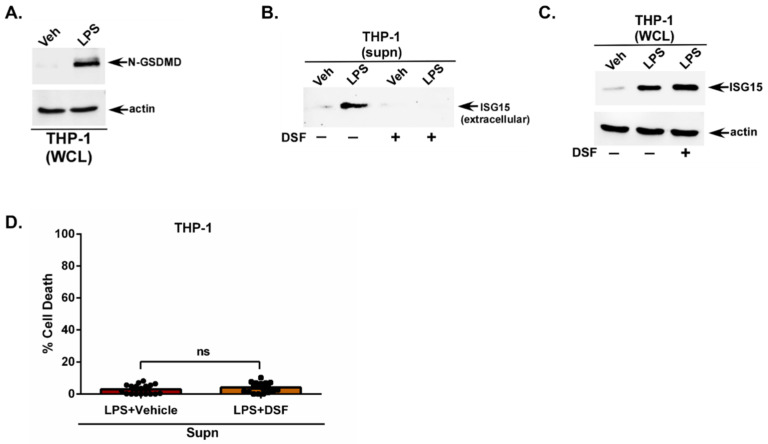
Gasdermin D (GSDMD) is involved during ISG15 release from human macrophages primed with extracellular LPS. (**A**) Human THP-1 macrophages were treated with either vehicle control (Veh) or LPS (1 µg/mL). Whole-cell lysate (WCL) was subjected to Western blotting with Gasdermin D (GSDMD) and actin antibodies. The cleaved N-terminal portion of GSDMD is shown in (**A**). (**B**) Medium supernatants (supn) from THP-1 macrophages treated with LPS in the presence of either GSDMD inhibitor disulfiram (DSF) (+) or vehicle control (−) were TCA-precipitated and analyzed using Western blotting with ISG15 antibody to detect extracellular ISG15 protein. (**C**) WCL from THP-1 macrophages treated with LPS in the presence of either DSF (+) or vehicle control (−) was subjected to Western blotting with ISG15 and actin antibodies to detect intracellular unconjugated ISG15 protein. (**D**) Medium supernatants (supn) from THP-1 macrophages treated with LPS in the presence of either DSF (+) or vehicle control (−) were used to quantify cell death using a lactate dehydrogenase (LDH) assay. The Western blots are representative of three independent experiments with similar results. ns; non-significant using a Student’s *t*-test.

Mechanistically, we postulated that GSDMD pores may be involved in the extracellular release of ISG15 from non-lytic cells following LPS stimulation since one study has reported GSDMD cleavage by LPS in murine macrophages [46]. Furthermore, this study also showed LPS-mediated GSDMD cleavage occurring only in the presence of high glucose in the culture medium [46]. Since GSDMD cleavage by LPS is unknown in human macrophages, we first evaluated the GSDMD cleavage status in LPS-treated human THP-1 macrophages. This experiment was performed in the presence of normal glucose levels in the culture medium. GSDMD cleavage status was monitored by performing Western blotting with an GSDMD antibody. Similarly to murine macrophages, LPS also triggered GSDMD cleavage in human macrophages as shown by the detection of cleaved N-terminal fragments of GSDMD in LPS-treated human THP-1 macrophages (Figure 2A). In contrast to murine macrophages, we detected the LPS-mediated cleavage of GSDMD in human macrophages when the glucose levels in the culture medium were normal.

Next, we used a well-known GSDMD inhibitor disulfiram (DSF) to assess the role of GSDMD during ISG15 release from LPS-stimulated macrophages. DSF blocks the formation of GSDMD pores on the cell surface [47]. The medium supernatant from THP-1 macrophages treated with LPS in the presence of either the vehicle (control) or DSF was analyzed through TCA precipitation and Western blotting with an ISG15 antibody. GSDMD inhibition resulted in a drastic loss of ISG15 release from LPS-treated cells (Figure 2B). The absence of ISG15 in the medium supernatant of LPS + DSF-treated cells is not due to a lack of intracellular ISG15, since Western blotting of the WCL with an ISG15 antibody detected an abundant intracellular expression of ISG15 protein in cells treated with LPS + DSF (Figure 2C). Moreover, the effect of the GSDMD inhibitor is independent of cellular cytotoxicity, since the treatment of THP-1 macrophages with the same concentration of the inhibitor (LPS + vehicle and LPS + DSF) did not affect cell viability as analyzed using the LDH assay (Figure 2D). Very minimal cell death (2–3.8% cell death) was observed in LPS-treated cells in the absence (vehicle-treated) and presence of the GSDMD inhibitor DSF (Figure 2D). These results have identified GSDMD as a critical cellular factor involved in the extracellular release of unconjugated free ISG15 protein.

### 3.3. Caspase-4 (Casp4) Is Required for Gasdermin D Cleavage and ISG15 Release from LPS-Stimulated Macrophages

A non-canonical inflammasome activation in murine macrophages by extracellular LPS has been reported whereby Casp11 (Casp4 and Casp5 in humans) cleaves GSDMD to generate the active pore-forming N-terminal fragment of GSDMD [48,49,50]. Therefore, we next examined whether Casp4 plays a role in LPS-mediated ISG15 release from human macrophages via GSDMD cleavage.

**Figure 3:**
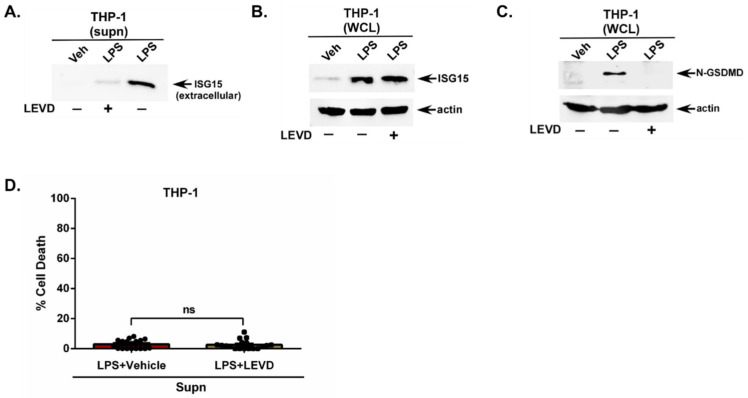
Caspase-4 (Casp4) is essential for ISG15 release from human macrophages primed with extracellular LPS. (**A**) Medium supernatants (supn) from human THP-1 macrophages treated with LPS in the presence of either caspase-4 (Casp4) inhibitor Ac-LEVD-CHO (LEVD) (+) or vehicle control (−) were TCA-precipitated and analyzed through Western blotting with ISG15 antibody to detect extracellular ISG15 protein. (**B**) Whole-cell lysate (WCL) from THP-1 macrophages treated with LPS in the presence of either LEVD (+) or vehicle control (−) was subjected to Western blotting with ISG15 and actin antibodies to detect intracellular unconjugated ISG15 protein. (**C**) THP-1 macrophages were treated with LPS in the presence of either LEVD (+) or vehicle control (−). WCL was subjected to Western blotting with Gasdermin D (GSDMD) and actin antibodies. The cleaved N-terminal portion of GSDMD is shown in (**C**). (**D**) Medium supernatants (supn) from THP-1 macrophages treated with LPS in the presence of either LEVD (+) or vehicle control (−) were used to quantify cell death using a lactate dehydrogenase (LDH) assay. The Western blots are representative of three independent experiments with similar results. ns; non-significant using a Student’s *t*-test.

For these studies, we utilized the cell-permeable Casp4 inhibitor Ac-LEVD-CHO (LEVD). This inhibitor blocks Casp4 activity by binding to its active site, thereby preventing substrate processing and blocking downstream signaling including GSDMD cleavage [51]. Human THP-1 macrophages were treated with LPS in the presence of either the vehicle control or LEVD. Western blotting of the TCA-precipitated medium supernatant revealed a loss in extracellular ISG15 release from LPS-treated macrophages in the presence of LEVD (Figure 3A). However, low levels of ISG15 remained detectable in the supernatant (Figure 3A), which may be attributable to the Casp5-mediated release of ISG15, since LEVD is specific for Casp4 but not Casp5. The lack of ISG15 in the medium supernatant of LPS + LEVD-treated cells is not due to insufficient intracellular ISG15 levels, since Western blotting of the WCL with an ISG15 antibody detected the intracellular expression of ISG15 protein in cells treated with LPS + LEVD (Figure 3B).

Since Casp4 acts upstream of GSDMD, we next evaluated the GSDMD status in cells treated with the Casp4 inhibitor LEVD. Western blotting of WCL with a GSDMD antibody demonstrated a drastic loss of GSDMD cleavage following Casp4 inhibition with LEVD (Figure 3C). Moreover, Casp4 inhibitor-mediated activity was independent of cellular cytotoxicity, since THP-1 macrophages treated with the same concentration of the inhibitor (LPS + vehicle and LPS + LEVD) had no effect on cell viability (Figure 3D). Cell death was very minimal (1.7–2.3% cell death) in LPS-treated cells in the absence (vehicle-treated) and presence of the Casp4 inhibitor (Figure 3D). Thus, we have identified the non-canonical Casp4 pathway as an important player promoting the release of unconjugated ISG15 from LPS-stimulated human macrophages.

### 3.4. Paracrine/Autocrine Action of Type-I Interferon (IFN) Is Involved in Extracellular LPS-Mediated ISG15 Release from Macrophages

Internalized LPS and cytoplasmic LPS delivered by Gram-negative bacteria activates the non-canonical inflammasome pathway in human macrophages [49,52]. Our current studies demonstrated another role of the non-canonical inflammasome in mediating ISG15 release from human macrophages stimulated with extracellular LPS. In previous studies, a non-canonical inflammasome activation by extracellular LPS was observed in murine macrophages, although the mechanism was unknown [48]. To unfold the mechanism driving extracellular LPS-mediated non-canonical inflammasome-dependent ISG15 release, we focused on the potential role of the paracrine/autocrine activity of type-I interferon (IFN). We chose to examine the involvement of IFN since IFN is produced from extracellular LPS-primed macrophages [53,54,55] and IFN activates the non-canonical inflammasome pathway in mouse macrophages by activating Casp11, the murine homolog of human Casp4/Casp5 [48]. We envisioned the paracrine/autocrine activity of IFN produced from LPS-primed macrophages triggering a non-canonical inflammasome-mediated ISG15 release via GSDMD pores.

**Figure 4:**
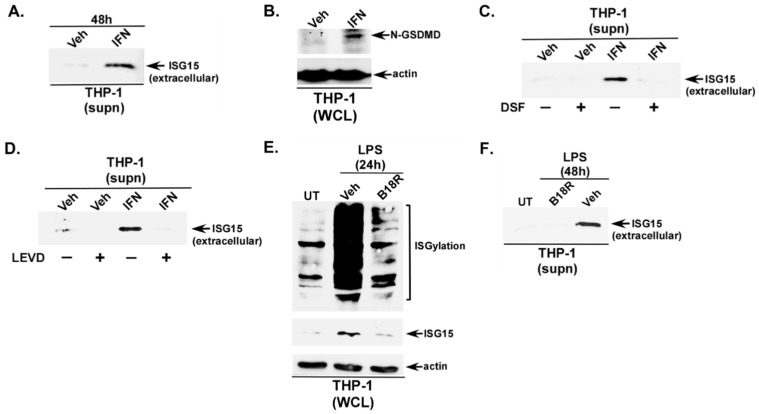
Paracrine/autocrine activity of type-I interferon triggers ISG15 release from human macrophages primed with extracellular LPS. (**A**) Human THP-1 macrophages were treated with either vehicle control (Veh) or IFN-β (IFN) (500 U/mL). The medium supernatants (supn) were TCA-precipitated and analyzed through Western blotting with ISG15 antibody to detect extracellular ISG15 protein. (**B**) THP-1 macrophages were treated with either vehicle control (Veh) or IFN. Whole-cell lysate (WCL) was subjected to Western blotting with Gasdermin D (GSDMD) and actin antibodies. The cleaved N-terminal portion of GSDMD is shown in (**B**). (**C**) Medium supernatants (supn) from THP-1 macrophages treated with IFN in the presence of either GSDMD inhibitor disulfiram (DSF) (+) or vehicle control (−) were TCA-precipitated and analyzed through Western blotting with ISG15 antibody to detect extracellular ISG15 protein. (**D**) Medium supernatants (supn) from human THP-1 macrophages treated with IFN in the presence of either caspase-4 (Casp4) inhibitor Ac-LEVD-CHO (LEVD) (+) or vehicle control (−) were TCA-precipitated and analyzed through Western blotting with ISG15 antibody to detect extracellular ISG15 protein. (**E**) WCL from untreated (UT) THP-1 macrophages or THP-1 cells treated with LPS in the presence of either type-I interferon inhibitor B18R or vehicle control (Veh) was subjected to Western blotting with ISG15 and actin antibodies to detect ISGylation and intracellular unconjugated ISG15 protein. (**F**) Medium supernatants (supn) from untreated (UT) and THP-1 macrophages treated with LPS in the presence of either B18R or vehicle control (Veh) were TCA-precipitated and analyzed through Western blotting with ISG15 antibody to detect extracellular ISG15 protein. The Western blots are representative of three independent experiments with similar results.

Although IFN (e.g., IFN-β) can trigger ISG15 release from various immune cells (PBMCs, T cells, monocytes) [16], IFN-mediated ISG15 release in human macrophages is not well studied. Specifically, the mechanism by which IFN triggers an unconjugated ISG15 release from macrophages is unknown. Thus, we performed a series of studies to decipher the role of the Casp4-GSDMD pathway in promoting IFN-mediated ISG15 release. Robust ISGylation and ISG15 expression were observed in IFN-β-treated THP-1 macrophages (Figure S2A). TCA precipitation of the medium supernatant also revealed unconjugated ISG15 release from IFN-treated macrophages (Figure 4A). Since GSDMD cleavage by LPS was involved in ISG15 release, we next evaluated the role of GSDMD in ISG15 release from IFN-treated macrophages. The activation of GSDMD by IFN was evident from the GSDMD cleavage in IFN-β-treated macrophages (Figure 4B). Furthermore, this cleavage was important for IFN-β-mediated ISG15 release, since a loss of ISG15 release was observed in macrophages treated with the GSDMD inhibitor DSF (Figure 4C). IFN also requires Casp4 for ISG15 release, since the treatment of THP-1 macrophages with Casp4 inhibitor LEVD drastically diminished ISG15 release from IFN-β-treated cells (Figure 4D). The lack of ISG15 in the medium supernatant of IFN-treated cells in the presence of either DSF or LEVD is not due to insufficient levels of intracellular ISG15, since Western blotting of the WCL with an ISG15 antibody detected the intracellular expression of ISG15 protein in cells treated with both IFN + DSF (Figure S2B) and IFN + LEVD (Figure S2C). These results demonstrated that IFN possess unconjugated ISG15 releasing activity, and this process is mediated via the Casp4-GSDMD pathway.

Since IFN triggered ISG15 release from human THP-1 macrophages via the Casp4-GSDMD pathway (Figure 4A–D) and LPS is a well-known producer of IFN from macrophages [53,54,55], we next examined whether the paracrine/autocrine action of IFN produced from LPS-primed macrophages is involved in ISG15 release. For these studies, we utilized B18R, a potent inhibitor of the IFN pathway. B18R has been widely used to block the activity of extracellular IFN [56]. Specifically, B18R binds with IFN to prevent its interaction with IFN receptors for the activation of the JAK/STAT pathway [56,57]. B18R is a potent inhibitor of IFN-mediated pro-ISGylation activity, since the treatment of THP-1 macrophages with IFN-β in the presence of a non-cytotoxic dose of B18R [58] abrogated ISGylation and ISG15 expression (Figure S3A). Thus, we next utilized B18R to investigate the autocrine/paracrine role of IFN in triggering ISGylation, ISG15 expression, and ISG15 release from extracellular LPS-primed macrophages. The autocrine/paracrine activity of IFN plays a major role in extracellular LPS-mediated activity since a drastic reduction in ISGylation and ISG15 expression was observed in macrophages treated with B18R (Figure 4E). Concomitantly, both GSDMD cleavage (Figure S3B) and ISG15 release (Figure 4F) from LPS-primed macrophages was diminished in the presence of B18R.

IFN confers its activity by activating the JAK/STAT pathway. Therefore, the role of IFN was further validated by using JAK inhibitor Ruxolitinib. The treatment of LPS-primed macrophages with a non-cytotoxic dose of Ruxolitinib [59] drastically diminished LPS-mediated ISG15 release (Figure S3C). This result confirmed the role of IFN during ISG15 release by extracellular LPS.

A previous study has implicated a role of autophagy in ISG15 release from virus-infected macrophages [26]. In order to investigate whether autophagy also plays a role in ISG15 release from LPS-primed macrophages, we treated the cells with the autophagy inhibitor 3-Methyladenine (3MA). Autophagy is not involved, since the treatment of cells with a non-cytotoxic dose of 3MA [60] did not affect ISG15 release from LPS-treated THP-1 macrophages (Figure S3D). Thus, our studies have highlighted a critical involvement of the autocrine/paracrine activity of IFN produced from LPS-primed macrophages in promoting ISGylation and ISG15 expression and subsequent ISG15 release via the Casp4-GSDMD pathway.

## 4. Discussion

ISGylation represents an important post-translational modification controlling a wide spectrum of biological and cellular functions/activities. During ISGylation, cytosolic ISG15 proteins undergo a covalent conjugation with target proteins [11,12,13,14]. Although the majority of the intracellular ISG15 protein is recruited for ISGylation, some of the unconjugated intracellular ISG15 protein is released to the extracellular milieu following the stimulation of cells with various external factors such as viral (SARS-CoV-2, influenza A virus, rhinovirus) infection and the activation of Pattern Recognition Receptors (PRRs like TLR1/2 and TLR3) by their corresponding Pathogen Associated Molecular Patterns (PAMPs—Pam3CSK4 and poly-I:C) [16,24,25,26,27,30]. The clinical relevance of extracellular ISG15 is evident from the detection of extracellular ISG15 proteins in the saliva, serum, and nasal secretion of SARS-CoV-2- and rhinovirus-infected patients [25,26,27]. More importantly, the levels of extracellular ISG15 in virus-infected patients were positively correlated with the viral load and clinical symptom score associated with the infection [25].

Functionally, extracellular ISG15 possesses immune-modulatory properties, since it regulated the production of the cytokine, interferon-γ (IFN-γ), and chemokine, CXCL10, from immune cells (NK cells, PBMCs, T cells) and non-immune epithelial cells, respectively [25,28,31,32]. One study reported that ISG15 release from virus-infected cells may occur via an autophagy-driven unconventional LC3-derived secretory pathway [26]. However, in our study, blocking autophagy had no effect on ISG15 release from LPS-primed human macrophages (Figure S3D). These results suggest that a distinct ISG15 release mechanism may exist during virus-infected vs. LPS-primed macrophages. In this scenario, the stimulus-dependent mechanism triggering ISG15 release may vary according to the nature of the stimulus (e.g., virus-infected vs. LPS-primed cells) and cellular signaling pathways involved during the specific stimuli. Specifically, while autophagy-dependent ISG15 release may predominate during viral infection, our study supports the involvement of a non-canonical inflammasome-dependent pathway in ISG15 release during LPS stimulation. Thus, it is imperative to identify the cellular mechanism(s) responsible for the extracellular release of ISG15, since ISG15 release occurs during various biological conditions via various stimuli. In the current study, we have utilized LPS as an ISG15 release stimulator in human macrophages to identify the pathway involved in the extracellular release of ISG15. Our studies demonstrated that, during extracellular LPS stimulation, macrophages trigger ISG15 release via the autocrine/paracrine activity of type I interferon (IFN). Specifically, IFN produced from LPS-treated cells results in Casp4-dependent GSDMD cleavage to form plasma membrane GSDMD pores, resulting in the extracellular release of ISG15 proteins. Thus, the IFN-Casp4-GSDMD pathway constitutes the first report of a cellular mechanism involved in the extracellular release of unconjugated ISG15 from cells.

Extracellular LPS is a major “endotoxin” PAMP involved in triggering exaggerated inflammation-driven septic shock (sepsis) in patients infected with clinically important Gram-negative bacteria such as *Escherichia coli*, *Klebsiella pneumoniae*, and *Pseudomonas aeruginosa* [35,37,38,61,62]. Additionally, although LPS is a major PAMP, no previous studies have investigated whether LPS can trigger ISG15 release. Moreover, LPS-mediated ISGylation has not been documented in human macrophages. Based on these knowledge gaps, we explored the ability of LPS to trigger ISGylation and ISG15 release from human THP-1 macrophages. Our study shows LPS promoting ISGylation and ISG15 release from human macrophages.

To elucidate the underlying mechanism for ISG15 release from LPS-stimulated cells, we focused on two cellular factors, Casp4 and GSDMD. Firstly, we focused on GSDMD, since cell surface GSDMD pores are utilized for the shuttling of ions and host factors, including cytokines like IL-18 and IL-1β [42]. The cleavage of GSDMD by either canonical (i.e., human/murine caspase-1) or non-canonical (i.e., human caspase-4/5, caspase-11 is the murine homolog of caspase-4/5) caspases results in the generation of active N-terminal fragments of GSDMD. These N-terminal fragments oligomerize to form GSDMD pores on the plasma membrane [42,63]. The activation and assembly of GSDMD pores is rapid after stimulation by various agents, and this process ensures the ability of GSDMD pores to shuttle proteins for extracellular release from intact non-lytic cells prior to the occurrence of the pyroptotic lytic cell death of macrophages, which occurs later due to the massive ionic imbalance that culminates in osmotic cell swelling and lysis [44,64]. For example, GSDMD pores are involved in the extracellular release of cytoplasmic proteins such as IL-1β and IL-18 from non-lytic cells [44]. The non-lytic release of IL-1β and IL-18 occurred due to the GSDMD pore permitting molecules up to 20 kDa–50 kDa to pass through the pore for extracellular release [44,65]. Our results revealed that small proteins like ISG15 (17 kDa) can also utilize GSDMD pores for their release, since blocking GSDMD pore formation drastically impaired ISG15 release from LPS-stimulated cells.

Both canonical and non-canonical inflammasome pathways can cleave GSDMD to generate the active pore-forming N-terminal fragment of GSDMD [49]. The canonical inflammasome activates caspase-1 via inflammasomes such as the NLRP3 inflammasome. It is unlikely that extracellular LPS is utilizing a canonical inflammasome pathway for triggering ISGylation, ISG15 release, and GSDMD cleavage, since second signals (e.g., potassium efflux, ATP, nigericin, ROS, etc.) are required for the extracellular LPS-mediated activation of the canonical inflammasome in macrophages [42,49,66,67]. Therefore, we focused our studies on the non-canonical inflammasome. The non-canonical inflammasome comprises human Casp4 (Casp11 in mice) and human Casp5 that cleaves GSDMD to generate the active pore-forming N-terminal fragment of GSDMD [49]. The LPS-TLR4 complex from the cell surface is internalized in myeloid cells like monocytes, macrophages, and DCs [68]. Upon internalization, LPS is released in the cytoplasm, which then interacts with Casp4/5 to activate the non-canonical inflammasome pathway. Alternatively, internalized Gram-negative bacteria deliver LPS in the cytoplasm to activate the Casp4/5 pathway [63]. However, in contrast to internalized LPS (i.e., cytoplasmic LPS), our results demonstrated extracellular LPS triggering ISGylation, ISG15 release, and GSDMD cleavage. In that context, non-canonical inflammasome activation in murine macrophages by extracellular LPS has been reported [48]. In this study, extracellular LPS activated Casp11, the murine homolog of human Casp4 and Casp5. Additionally, GSDMD cleavage in murine macrophages was observed following stimulation with extracellular LPS in a high-glucose medium [46]. We now show LPS triggering GSDMD cleavage in human macrophages, which in contrast to murine macrophages occurred in a normal-glucose medium. These glucose level-dependent differences in human and murine macrophages may reflect a species-specific metabolic regulation of non-canonical inflammasome signaling. This species-specific phenomenon may occur due to glucose regulating (a) the expression/activity of non-canonical inflammasome pathway components such as Casp4, GSDMD, and/or (b) the threshold requirement for GSDMD cleavage by caspases. In this scenario, non-canonical inflammasome-mediated GSDMD cleavage in murine macrophages may only occur in the presence of high glucose in the medium, whereas human macrophages can trigger the same pathway in the presence of normal glucose levels in the medium. Interestingly, the inhibition of Casp4 led to a reduced GSDMD cleavage and ISG15 release from human macrophages treated with extracellular LPS. Thus, our studies have highlighted a role of non-canonical inflammasome apparatus like Casp4 for GSDMD cleavage and ISG15 release from human macrophages stimulated with extracellular LPS.

To decipher the mechanism of extracellular LPS-mediated non-canonical inflammasome activation, we focused on the possible role of type I interferon (IFN), since extracellular LPS is known to produce IFN after the activation of TLR4 in human macrophages and because, more importantly, the activation of the non-canonical inflammasome by IFN in macrophages has been reported [48]. It is postulated that extracellular IFN interacts with the cell surface IFN receptor to activate the JAK-STAT pathway for non-canonical inflammasome activation [69]. Our studies utilized potent IFN pathway inhibitor B18R to demonstrate that the paracrine/autocrine activity of IFN produced from extracellular LPS-primed macrophages is involved in triggering non-canonical inflammasome-mediated ISG15 release via pores formed by cleaved GSDMD. We show IFN utilizing the non-canonical Casp4/GSDMD inflammasome pathway for ISG15 release from human macrophages.

In the context of Gram-negative bacterial sepsis, the LPS-mediated extracellular release of ISG15 from macrophages via the Casp4-GSDMD pathway identified in this study may have important pathophysiological implications. Extracellular ISG15 has been reported to function as a cytokine-like immunomodulator that acts on NK cells and T lymphocytes to promote proliferation and IFN-γ secretion [20]. Therefore, we postulate that, during the early phase of infection, the activation of NK and T cells can enhance pathogen clearance through IFN-γ production and the recruitment of other immune cells [70,71], suggesting that, at this stage, extracellular ISG15 may contribute to anti-bacterial immunity. However, an excessive or sustained activation of these immune cells can amplify systemic inflammation and worsen disease outcomes, which culminates in sepsis [72]. Thus, as sepsis progresses, widespread T-cell dysfunction drives the immunosuppressive phase of the disease [73], which may potentially shift the “beneficial” protective anti-bacterial effect of extracellular ISG15 toward “detrimental” immune dysregulation/suppression. Although the exact role of extracellular ISG15 during sepsis needs to be investigated in the future, our findings provide a mechanistic framework linking extracellular ISG15 release from LPS-primed macrophages to its potential role in maintaining a dynamic balance between anti-bacterial immune response, inflammation, and immunosuppression during Gram-negative sepsis.

Non-canonical inflammasome activation in human (Casp4, Casp5) and mouse (Casp11) cells results in GSDMD activation [49]. In that regard, the activation of Casp11 in murine macrophages by extracellular LPS has been reported [48]. Following activation, Casp4, Casp5, and Casp11 undergo autocleavage for their activation, leading to GSDMD cleavage. Since the autocleavage mechanisms of Casp4 are better characterized than Casp5, in the current study, we chose to focus on the role of Casp4 during extracellular LPS-mediated ISG15 release [42]. In the future, we will study whether Casp5 along with Casp4 is required for GSDMD-mediated ISG15 release. This possibility is supported by our observation that the inhibition of Casp4 reduced, but did not completely eliminate, ISG15 release from LPS-treated human macrophages, suggesting that additional non-canonical inflammasome components like Casp5 may contribute to this process. Furthermore, ISG15 release from non-immune cells like epithelial cells has been documented during various conditions including viral infections and cancer [74]. In contrast to immune myeloid cells like macrophages, non-immune/non-myeloid cells such as epithelial cells predominantly express GSDME. GSDME undergoes cleavage by Casp3 [75], and the resulting cleaved N-terminal fragment of GSDME forms plasma membrane pores similar to cleaved GSDMD [76]. Thus, we postulate that, like GSDMD in immune myeloid cells (macrophages), GSDME in non-myeloid cells (epithelial cells) may be involved in ISG15 release during various conditions. In that regard, a cell type-specific alternative ISG15 release pathway (e.g., Casp3-GSDME) may exist in epithelial cells compared to the Casp4/5-GSDMD pathway in macrophages. Therefore, in the future, we will focus on the role of Casp3 and GSDME in facilitating ISG15 release from non-immune/non-myeloid cells like epithelial cells.

**Figure 5:**
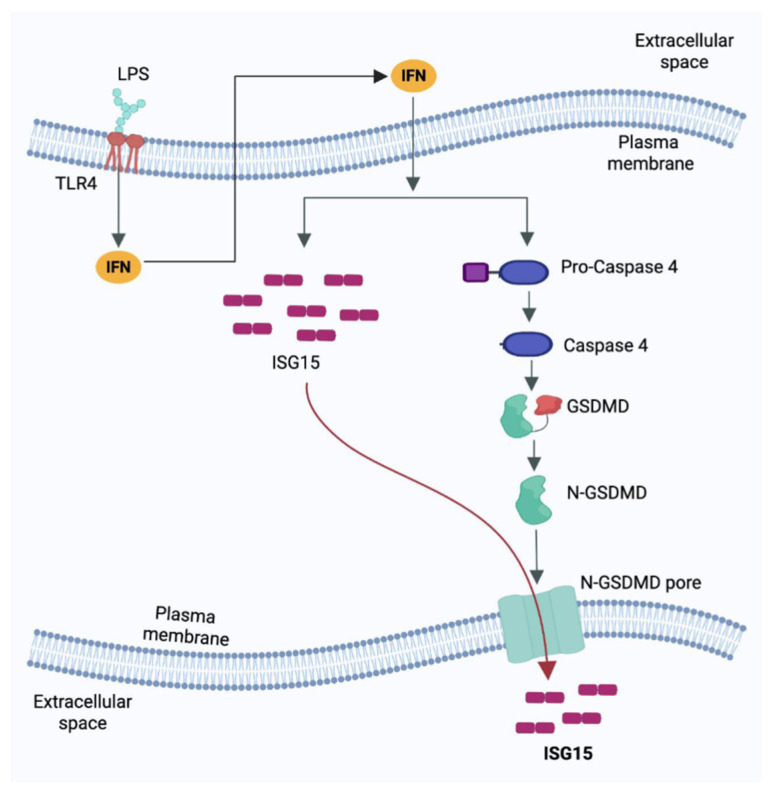
Schematic model showing the extracellular release mechanism of unconjugated “free” ISG15 protein from human macrophages stimulated with lipopolysaccharide (LPS). Extracellular LPS interacts with toll-like receptor 4 (TLR4) to activate the TLR4 pathway leading to production of type-I interferons (IFN). Paracrine/autocrine action of the extracellularly released IFN triggers expression of ISG15 in target cells. Subsequently, pro-caspase-4 cleavage by IFN action leads to its activation, and cleavage of the Gasdermin-D (GSDMD) protein. The cleaved N-terminal portion of GSDMD (N-GSDMD) forms pores on the plasma membrane. The intracellular unconjugated ISG15 utilizes cell surface GSDMD pored for its release into the extracellular milieu.

In summary, our current study is the first report of a mechanism involved in the extracellular release of unconjugated ISG15, which occurred in non-lytic cells via the Casp4-GSDMD cellular pathway (Figure 5). The engagement of LPS with TLR4 results in activation of the TLR4 pathway, which culminates in the production of type-I interferons (IFN). Extracellular IFN via a paracrine/autocrine mechanism interacts with the cell surface IFN receptor to upregulate the expression of interferon-stimulated genes (ISGs) such as ISG15. Simultaneously, IFN signaling also activates Casp4, which then cleaves GSDMD to facilitate the formation of GSDMD pores on the plasma membrane. The GSDMD pores are utilized to release unconjugated ISG15 into the extracellular milieu (Figure 5). Collectively, this mechanism highlights the interplay between LPS-TLR4-IFN signaling and the Casp4-GSDMD pathway in triggering ISG15 release from human macrophages.

## Data Availability

The original contributions presented in this study are included in the article/Supplementary Materials. Further inquiries can be directed to the corresponding author.

## Supplementary Materials

The following supporting information can be downloaded at https://www.mdpi.com/article/10.3390/pathogens15010122/s1. Figure S1: Extracellular LPS promotes ISG15 release from human THP-1 macrophages via TLR4 dependent pathway; Figure S2: Interferon-b triggers ISGylation and intracellular ISG15 expression in human THP-1 macrophages; Figure S3: Type-I interferon mediated JAK/STAT signaling is required for extracellular LPS mediated Gasdermin D cleavage and ISG15 release from human THP-1 macrophages. Autophagy is not involved during this process.
