# Supplementary material for: Extracellular Lipopolysaccharide Triggers the Release of Unconjugated Interferon-Stimulated Gene 15 (ISG15) Protein from Macrophages via Type-I Interferon/Caspase-4/Gasdermin-D Pathway
Source: Pathogens. 2026 Jan 22;15(1):122. doi: 10.3390/pathogens15010122 (PMC12844758; doi:10.3390/pathogens15010122)
Supplement: Supplementary file 1 [file pathogens-15-00122-s001.zip › pathogens-4035294-supplementary.pdf]

Extracellular Lipopolysaccharide triggers the release of unconjugated Interferon stimulated gene 15 (ISG15) protein from macrophages via Type-I interferon/Caspase-4/Gasdermin-D pathway

Supplementary Figure legends

Supplementary Figure S1. (A) Human THP-1 macrophages were treated with either vehicle control (Veh) or LPS (1µg/mL) for 48h and whole cell lysate (WCL) was subjected to western blotting with ISG15 and actin antibodies to detect ISGylation and intracellular unconjugated ISG15 protein. (B) Medium supernatants (supn) from THP-1 macrophages treated with LPS for 48h as described in Supplemental Fig. 1A were TCA-precipitated and analyzed by western blotting with ISG15 antibody to detect extracellular ISG15 protein. (C) Medium supernatants (supn) from human THP-1 macrophages treated with either vehicle control (Veh) or LPS (0.5µg/mL) in the presence of either TLR4 inhibitor TAK-242 (+) or TAK-242 vehicle control (-) were TCA-precipitated and analyzed by western blotting with ISG15 antibody to detect extracellular ISG15 protein. (D) Medium supernatants (supn) obtained from THP-1 macrophages treated with either vehicle control (Veh) or LPS were immune-precipitated (IP) with ISG15 antibody. The immune-precipitated proteins were subjected to western blotting with ISG15 antibody.

Supplementary Figure S2. (A) Human THP-1 macrophages were treated with either vehicle control (Veh) or IFN-β (IFN) (1000 IU/mL) for 24h. Whole cell lysate (WCL) was subjected to western blotting with ISG15 and actin antibodies to detect ISGylation and intracellular unconjugated ISG15 protein. (B) WCL from THP-1 macrophages treated with IFN (500 U/ml) in the presence of either GSDMD inhibitor disulfiram (DSF) (+) or DSF vehicle control (-) were subjected to western blotting with ISG15 and actin antibodies to detect intracellular unconjugated ISG15 protein. (C) WCL from human THP-1 macrophages treated with IFN (500 U/ml) in the presence of either Caspase 4 (Casp4) inhibitor Ac-LEVD-CHO (LEVD) (+) or LEVD vehicle control (-) were subjected to western blotting with ISG15 and actin antibodies to detect intracellular unconjugated ISG15 protein.

Supplementary Figure S3. (A) Whole cell lysate (WCL) from untreated (UT) THP-1 macrophages or THP-1 cells treated with IFN-β (IFN) (500 U/mL) in the presence of either type-I interferon inhibitor B18R or B18R vehicle control (Veh) was subjected to western blotting with ISG15 and actin antibodies to detect ISGylation and intracellular unconjugated ISG15 protein. (B) WCL from untreated (UT) THP-1 macrophages or THP-1 cells treated with LPS in the presence of either type-I interferon inhibitor B18R or B18R vehicle control (Veh) was subjected to western blotting with Gasdermin D (GSDMD) and actin antibodies. The cleaved N-terminal portion of GSDMD is shown in Supplementary Fig. 3B. (C) Medium supernatants (supn) from THP-1 macrophages treated with either vehicle control (Veh) or LPS in the presence of either JAK inhibitor Ruxolitinib (+) or Ruxolitinib vehicle control (-) were TCA-precipitated and analyzed by western blotting with ISG15 antibody to detect extracellular ISG15 protein. (D) Medium supernatants (supn) from THP-1 macrophages treated with either vehicle control (Veh) or LPS in the presence of either autophagy inhibitor 3MA (+) or 3MA vehicle control (-) were TCA-precipitated and analyzed by western blotting with ISG15 antibody to detect extracellular ISG15 protein.

Fig. S1

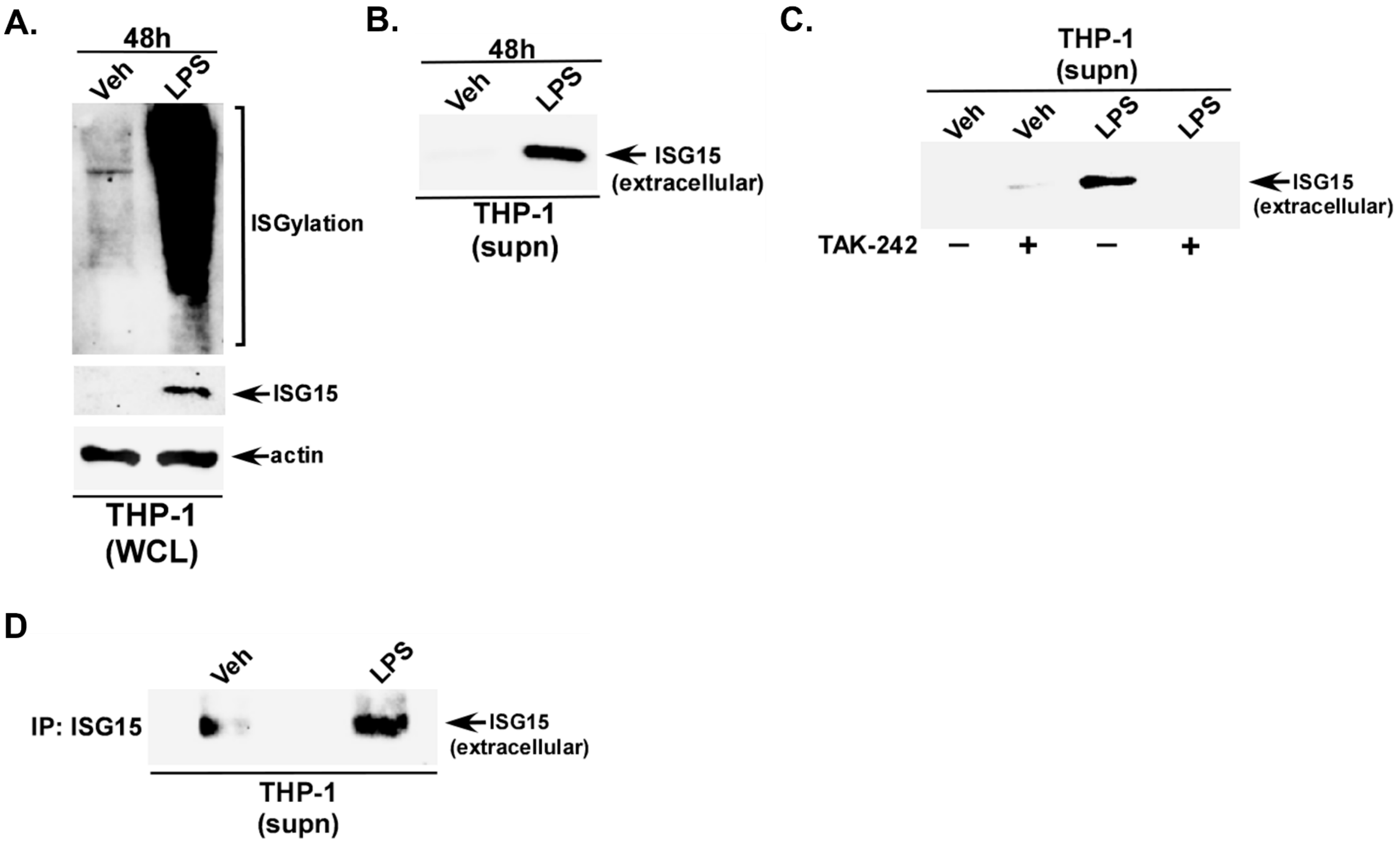
A.
48h
Veh
LPS
ISGylation
ISG15
actin
THP-1
(WCL)
B.
48h
Veh
LPS
ISG15
(extracellular)
THP-1
(supn)
C.
THP-1
(supn)
Veh
Veh
LPS
LPS
ISG15
(extracellular)
TAK-242
−
+
−
+
D
Veh
LPS
IP: ISG15
ISG15
(extracellular)
THP-1
(supn)

Fig. S2

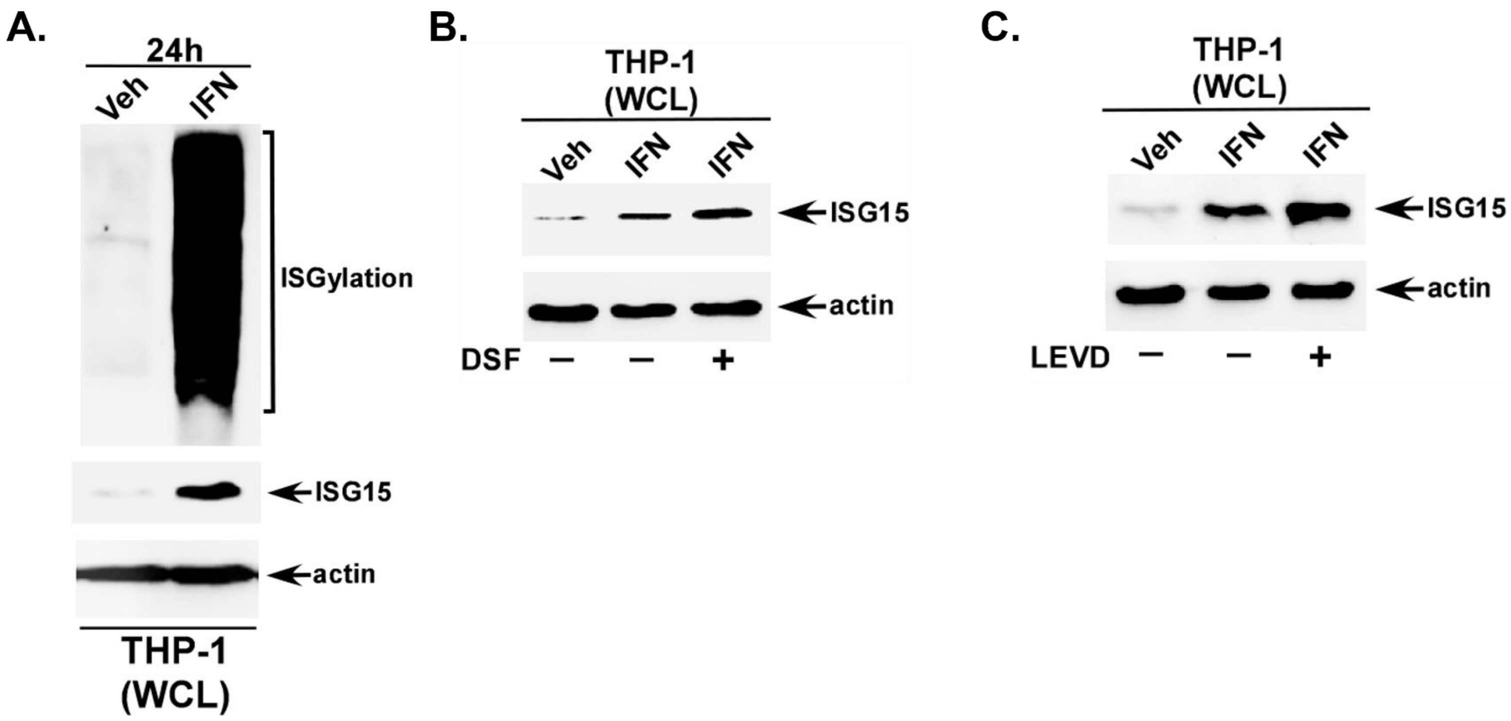
A.
24h
Veh
IFN
ISGylation
ISG15
actin
THP-1
(WCL)
B.
THP-1
(WCL)
Veh
IFN
IFN
ISG15
actin
DSF − − +
C.
THP-1
(WCL)
Veh
IFN
IFN
ISG15
actin
LEVD − − +

Fig. S3

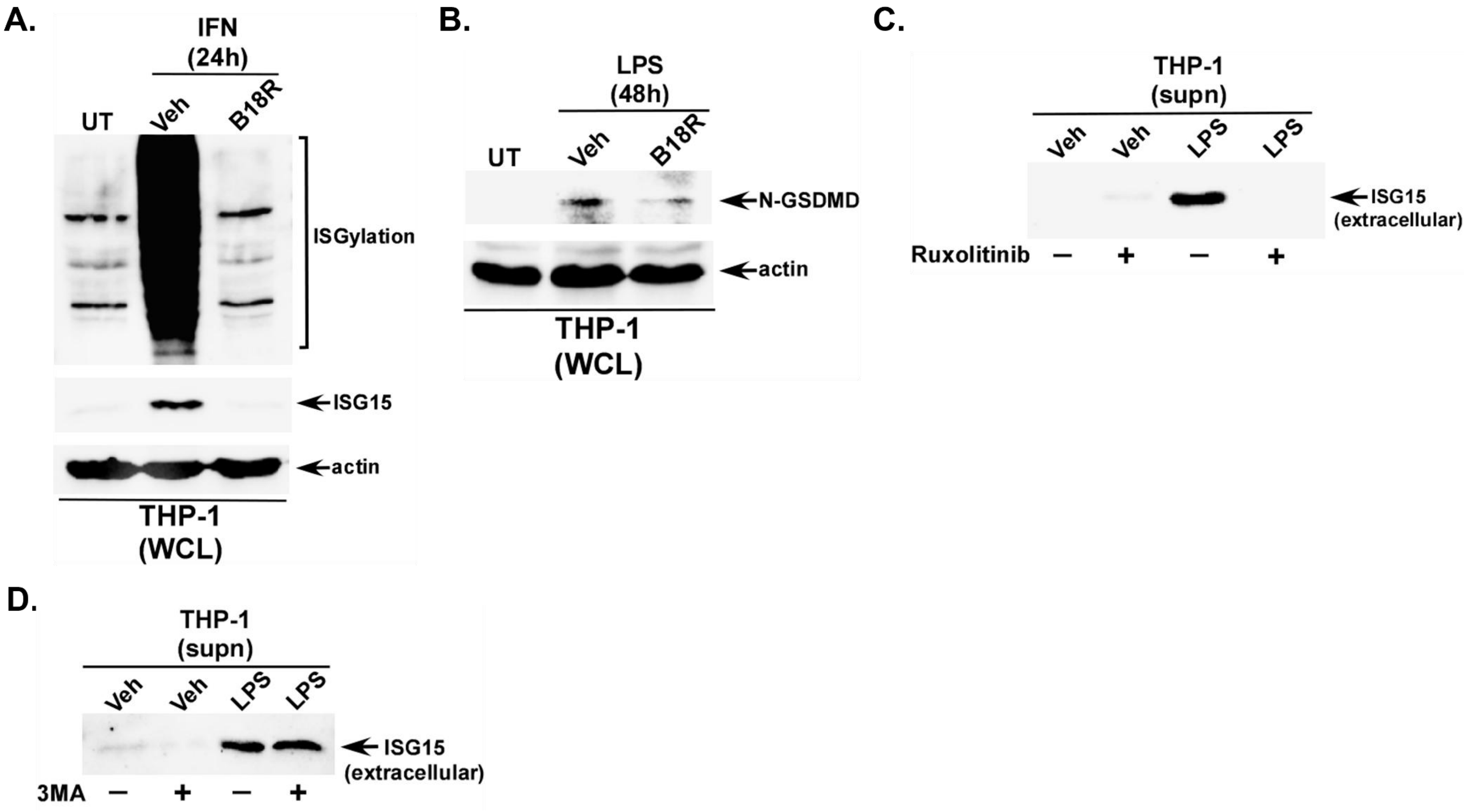
A.
IFN
(24h)
UT
Veh
B18R
ISGylation
ISG15
actin
THP-1
(WCL)
B.
LPS
(48h)
UT
Veh
B18R
N-GSDMD
actin
THP-1
(WCL)
C.
THP-1
(supn)
Veh
Veh
LPS
LPS
ISG15
(extracellular)
Ruxolitinib − + − +
D.
THP-1
(supn)
Veh
Veh
LPS
LPS
ISG15
(extracellular)
3MA − + − +
